# Serum Lipid Abnormalities in Pre-malignant and Malignant Lesions of the Gall Bladder

**DOI:** 10.7759/cureus.90229

**Published:** 2025-08-16

**Authors:** Lovely Singh, Syed Fiza Mustaqueem, Priyanka Singh, Pooja Jaiswal, Taj Mohd, Roshan Alam, Ashutosh Gupta, Rohit Keshav

**Affiliations:** 1 Department of Pathology, Integral Institute of Medical Sciences and Research, Lucknow, IND; 2 Department of Biochemistry, Integral Institute of Medical Sciences and Research, Lucknow, IND; 3 Department of Surgery, Integral Institute of Medical Sciences and Research, Lucknow, IND; 4 Department of Neurology, Institute of Neurosciences Kolkata, Kolkata, IND

**Keywords:** carcinogenesis, gallbladder cancer, hypertriglyceridemia, lipid metabolism, premalignant and malignant lesion

## Abstract

Background: Gallbladder pathology encompasses a spectrum from benign inflammation to aggressive malignancies, with gallbladder cancer (GBC) being one of the most lethal gastrointestinal cancers. Emerging evidence suggests that metabolic dysregulation, particularly lipid abnormalities, may drive carcinogenesis. However, the relationship between serum lipid profiles and gallbladder lesion progression remains poorly characterized.

Objective: This study aimed to investigate serum lipid abnormalities across benign (n = 48), premalignant (n = 11), and malignant (n = 12) gallbladder lesions to identify potential metabolic biomarkers for early detection and risk stratification.

Methods: A cross-sectional study was conducted on 71 cholecystectomy specimens (benign, premalignant, malignant) from April 2023 to March 2025. Histopathological evaluation categorized lesions, while fasting serum lipid profiles (total cholesterol, triglycerides, low-density lipoprotein (LDL), high-density lipoprotein (HDL), and very-low-density lipoprotein (VLDL)) were analyzed using automated biochemical assays. Statistical analysis included ANOVA and Pearson correlation tests, with significance at p < 0.05.

Results: The cohort (mean age: 48.7 ± 14.1 years) showed female predominance (77.5%). Malignant lesions presented at older ages (mean: 55.1 years) and exhibited significant hypertriglyceridemia (301.75 ± 85.69 mg/dL; p = 0.014) and elevated VLDL (54.82 ± 22.74 mg/dL; p = 0.052) compared to benign/premalignant groups. HDL was the lowest in malignancies (37.00 ± 9.05 mg/dL).

Conclusion: Progressive lipid derangements - particularly hypertriglyceridemia, elevated VLDL, and reduced HDL - are associated with malignant transformation in gallbladder lesions. Serum lipid profiling may serve as a non-invasive tool for risk stratification and early detection. These findings highlight the potential for metabolic interventions in high-risk populations, though further longitudinal studies are needed to establish causality and therapeutic implications.

## Introduction

Gallbladder pathology encompasses a broad spectrum of conditions, ranging from benign inflammatory disorders to aggressive malignancies, with significant geographical variations in disease prevalence and patterns. Gallbladder ranks among the most frequently resected organs worldwide, with chronic cholecystitis with cholelithiasis representing the most common pathological finding [1,2]. Gallstone disease affects 10-20% of the global adult population, according to WHO and large-scale epidemiological studies conducted in North America, Europe, and India between 2010 and 2021, and constitutes a well-established risk factor for gallbladder carcinogenesis [3]. The histopathological landscape of gallbladder disease includes reactive changes (cholesterolosis, xanthogranulomatous cholecystitis), premalignant lesions (adenomatous hyperplasia, metaplasia, dysplasia), and malignant transformation, particularly adenocarcinoma [2].

Gallbladder cancer (GBC) represents one of the most aggressive gastrointestinal malignancies, accounting for approximately 4% of digestive system cancers and nearly 50% of all biliary tract malignancies [4,5]. The disease demonstrates marked gender disparity, affecting women two to six times more frequently than men, with particularly high incidence rates along India's Gangetic belt [6-8]. The Indian Council of Medical Research reports age-adjusted incidence rates of 4.5 and 10.1 per 100,000 population for males and females, respectively, in northern India [8,9]. GBC carries a dismal prognosis, with five-year survival rates below 18%, largely attributable to advanced stage at diagnosis due to nonspecific early symptoms and the organ's deep anatomical location [8,10,11].

The carcinogenic sequence in gallbladder epithelium is postulated to progress through metaplastic, dysplastic, and carcinoma in situ stages before invasive malignancy develops [12,13]. Unlike colorectal carcinogenesis, where adenomatous polyps serve as common precursors, gallbladder adenomas are rare, suggesting alternative pathways for malignant transformation [14]. Current diagnostic modalities, including ultrasound, CT, and MRI, primarily detect structural changes and are limited in identifying early molecular alterations [15,16]. The absence of reliable biochemical markers for early detection underscores the need for novel diagnostic approaches [17].

Emerging evidence implicates metabolic dysregulation, particularly lipid metabolism abnormalities, in gallbladder carcinogenesis. Shared risk factors between cholesterol gallstones and GBC, including obesity, high-fat diets, and dyslipidemia, suggest potential common pathogenic mechanisms [7,18]. Epidemiological studies consistently demonstrate associations between hypertriglyceridemia, low high-density lipoprotein (HDL) levels, and GBC risk, though the relationship with total cholesterol and low-density lipoprotein (LDL) remains less clear [19,20]. The biological plausibility of this association stems from the role of lipid metabolites in cellular proliferation, membrane biosynthesis, and inflammatory signaling pathways that may drive the metaplasia-dysplasia-carcinoma sequence [21].

Despite these observations, the precise relationship between serum lipid profiles and epithelial proliferative activity across the spectrum of gallbladder lesions remains poorly characterized. This knowledge gap is particularly significant given the potential for serum lipid parameters to serve as accessible biomarkers for risk stratification and early detection. Furthermore, the proliferative characteristics of cholesterolosis - a condition characterized by abnormal lipid deposition in the gallbladder mucosa - remain underexplored in the context of its carcinogenic potential.

The current study aims to investigate the serum lipid abnormalities in premalignant and malignant gallbladder lesions. By finding specific lipid profile alterations (cholesterol, LDL, HDL, very-low-density lipoprotein (VLDL), and triglycerides) across the histological spectrum of gallbladder pathology, we seek to elucidate potential metabolic drivers of carcinogenesis. These insights may inform novel strategies for risk assessment, early detection, and targeted prevention in this aggressive malignancy with limited therapeutic options [22]. By clarifying the serum lipid profile alterations associated with gallbladder lesion progression, this study aims to provide insights that could inform the development of non-invasive metabolic biomarkers for early cancer detection and guide targeted preventive interventions.

Study objectives

The primary objective of this study was to investigate and compare serum lipid profiles among patients with benign, premalignant, and malignant gallbladder lesions, with the aim of identifying characteristic lipid abnormalities associated with progression along the gallbladder carcinogenesis spectrum. Specifically, we aimed to identify characteristic patterns of dyslipidemia associated with lesion progression, with the goal of evaluating the potential utility of serum lipid parameters as non-invasive metabolic biomarkers for early detection and risk stratification in GBC.

## Materials and methods

Study design and setting

A prospective cross-sectional observational study was conducted in the Department of Pathology at Integral Institute of Medical Science and Research (IIMSR), Lucknow, India, over a two-year period from April 2023 to March 2025.

Patient Selection

This study included all consecutive patients undergoing cholecystectomy at IIMS&R during the above-mentioned period who met the inclusion criteria. This consecutive sampling approach was adopted to minimize selection bias and comprehensively represent gallbladder lesions encountered during the study period. Although specimens were collected prospectively over two years, both clinical and biochemical data were analyzed at the time of surgery without follow-up, fulfilling the criteria for a cross-sectional analytic approach. The study protocol was approved by the Institutional Ethics Committee of the institute with IEC no. IEC/IIMSR/2023/55, dated 16 May 2023. Strict maintenance of patient confidentiality was ensured, and written informed consent was obtained from each participant.

Inclusion criteria comprised all gallbladder specimens showing benign (e.g., chronic cholecystitis and its variants), premalignant (e.g., metaplasia, dysplasia), or malignant changes, with complete clinical and biochemical data and adequate tissue, while exclusion criteria eliminated cases with neoadjuvant therapy, concurrent malignancies, inadequate samples, or missing data, ensuring robust and ethically compliant research methodology. This criterion reflects the inclusion of all consecutive cholecystectomy cases during the recruitment window, ensuring representation across the full histopathological spectrum for comparative analysis. The study utilized both archival and fresh gallbladder specimens obtained post-operatively from the Department of General Surgery, IIMSR.

A sample size of 71 patients was determined based on convenience sampling of all eligible cholecystectomy specimens collected consecutively over the two-year study duration. Formal power calculations were not performed, reflecting the exploratory nature of this research given sparse prior data on lipid abnormalities in gallbladder lesion progression.

Sample collection and processing

A total of 71 fresh cholecystectomy specimens demonstrating cholesterolosis, metaplastic, dysplastic, or malignant changes were collected in 10% neutral buffered formalin and processed following standard histopathological protocols, including fixation for 24-48 hours, gross examination, tissue processing through graded alcohols and xylene, paraffin embedding, sectioning at 4 μm thickness, and routine H&E staining.

Histopathological evaluation

All histopathological slides were independently reported by two experienced pathologists, both blinded to patients’ serum lipid results. Any discrepancies in lesion classification were resolved by joint consensus to ensure diagnostic consistency and minimize observer/classification bias. However, formal inter-observer agreement (κ statistic) was not calculated; this constitutes a limitation of our study.

Lesions were classified according to standard histopathological criteria: benign lesions included chronic cholecystitis, cholelithiasis, xanthogranulomatous cholecystitis, and cholesterolosis; premalignant lesions encompassed metaplasia and dysplasia; and malignant lesions included carcinoma in situ and invasive adenocarcinoma. Diagnostic criteria were applied consistently by pathologists following established guidelines cited from relevant authoritative sources.

Serum lipid analysis

Fasting venous blood samples (3 mL) were collected for serum lipid analysis using a Siemens Dimension RxL Max automated biochemical analyzer, measuring total cholesterol (CHOD-PAP method), triglycerides (GPO-PAP method), HDL cholesterol (PEG precipitation method), and calculated LDL cholesterol (Friedewald's equation). Routine calibration and internal quality control procedures were strictly adhered to during the study period to ensure data reliability. All blood samples for lipid profile estimation were collected pre-operatively on the morning of surgery (between 6:00 and 8:00 AM), following a minimum overnight fasting interval of ≥8 hours prior to any anesthesia or operative intervention. Fasting status was verbally confirmed with each patient on the morning of surgery before sample procurement to ensure comparability of serum lipid measurements. For cases with serum triglycerides >400 mg/dL, LDL was not calculated using the Friedewald formula due to its unreliability; these cases were labeled as "not available" for LDL and excluded from LDL subgroup analyses.

All specimens underwent comprehensive histopathological evaluation for diagnosis (benign, premalignant, malignant), specific pathological changes (metaplasia, dysplasia, malignancy), and cholesterolosis features.

Statistical analysis

Data were entered and analyzed using Microsoft Excel (Microsoft® Corp., Redmond, WA) and SPSS Statistical Product and Service Solutions (SPSS, version 25.0; IBM SPSS Statistics for Windows, Armonk, NY). Descriptive statistics were used to summarize demographic and clinicopathological variables. Mean and standard deviation (SD) were calculated for data set. For comparison of lipid parameters across different histopathological diagnoses, the normality of continuous data was assessed using the Shapiro-Wilk test, and homogeneity of variances was evaluated with Levene’s test. Where assumptions of homogeneity were not met, Welch’s ANOVA was employed instead of the standard ANOVA to compare lipid parameters across histopathological groups. Significant omnibus tests were followed by Games-Howell post-hoc comparisons to adjust for multiple testing without assuming equal variances. The correlation between lipid profile parameters across different histopathological categories was analyzed using Pearson’s correlation coefficient (r). A p-value <0.05 was considered statistically significant for all comparisons.

## Results

The study cohort comprised 71 patients with gallbladder lesions, demonstrating a mean age of 48.7±14.1 years with a striking female predominance (77.5%, n = 55), consistent with established epidemiological patterns of gallbladder disease. Age distribution analysis revealed peak incidence in the 51-60-year group (26.8%, n = 19) (Table 1), with malignant lesions presenting at significantly older ages (mean: 55.1 years) compared to premalignant (mean: 53.4 years) and benign lesions (mean: 46.0 years), suggesting an age-dependent progression in disease severity.

**Table 1:** Age group-wise distribution of cases

Age Group (years)	Number of Patients (n)	Frequency (%)
<30	9	12.68%
31–40	11	15.49%
41–50	15	21.13%
51–60	19	26.76%
61–70	14	19.72%
71–80	3	4.23%
Total	71	100%

**Figure 1:**
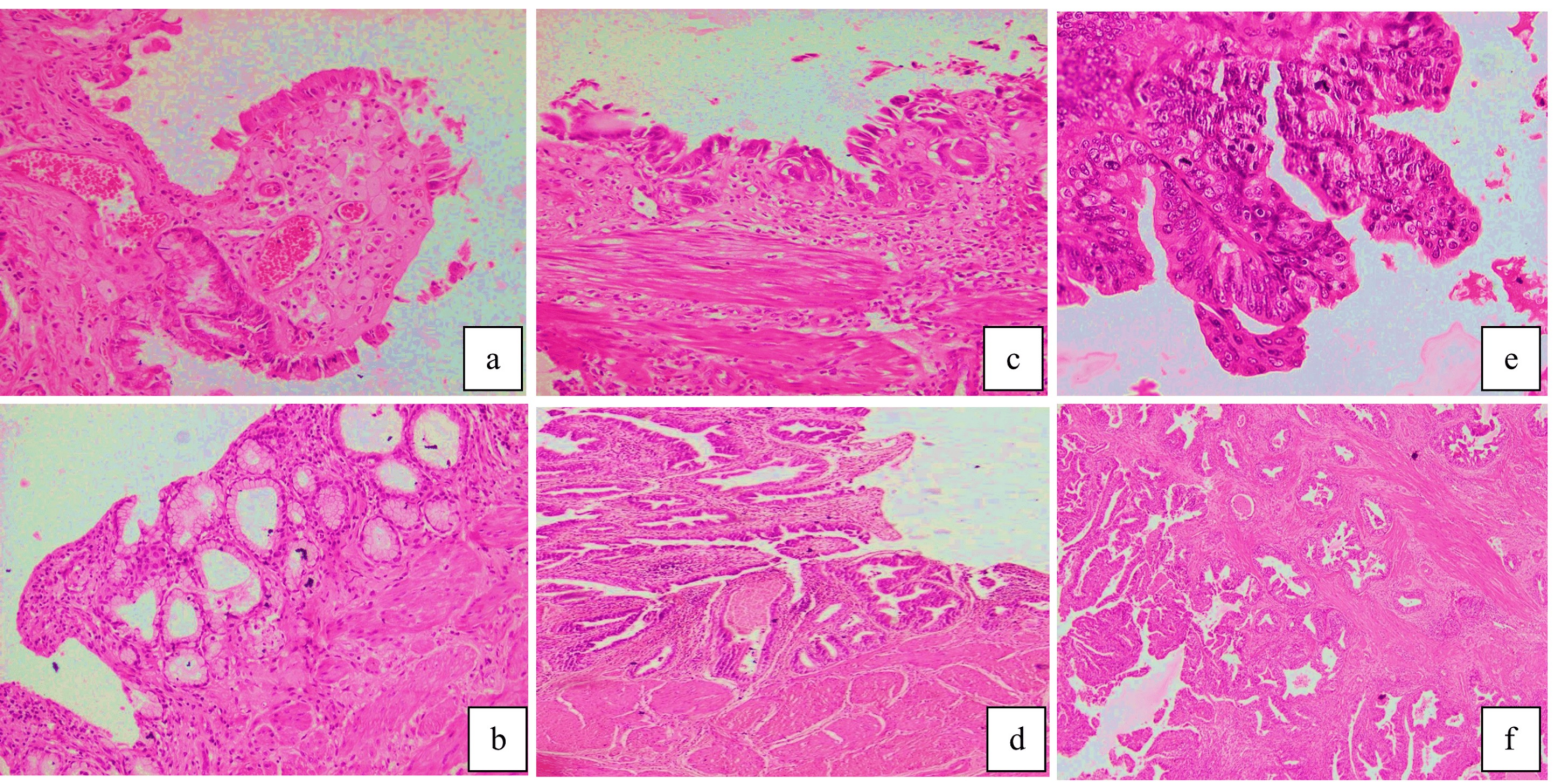
Microphotographs showing different histopathological lesions of the gall bladder (a) A case of cholesterolosis (H&E x 200x). (b) A case of chronic cholecystitis with metaplasia (H&E x 200x). (c) A case of dysplasia (H&E x 200x). (d) A case of carcinoma-in-situ (H&E x 50x). (e) A case of well-differentiated adenocarcinoma (H&E x 400x). (f) A case of moderately differentiated adenocarcinoma (H&E x 50x).

Histopathological examination categorized lesions into benign (n = 48, 67.6%), premalignant (n = 11, 15.5%), and malignant (n = 12, 16.9%) groups. The spectrum included chronic cholecystitis variants (with cholesterolosis, n = 12, 16.9%; with cholelithiasis, n = 10, 14.1%; with metaplasia, n = 9, 12.7%), xanthogranulomatous cholecystitis (n = 7, 9.9%), dysplasia (n = 11, 15.5%), and malignancies (adenocarcinoma, n = 10, 14.1%; carcinoma in situ (CIS), n = 2, 2.8%) (Table 2, Figure 1). Notably, malignant and premalignant lesions showed distinct clustering in older age groups (61-70 and 51-60 years, respectively), while benign lesions predominated in younger patients (41-50 years).

**Table 2:** Distribution of cases based on histopathological diagnosis

Histopathological Diagnosis	Number of Cases (n)	Percentage (%)
Chronic cholecystitis with cholesterolosis	12	16.90%
Dysplasia	11	15.49%
Chronic cholecystitis	10	14.08%
Chronic cholecystitis with cholelithiasis	10	14.08%
Adenocarcinoma	10	14.08%
Chronic cholecystitis with metaplasia	9	12.68%
Xanthogranulomatous cholecystitis	7	9.86%
CIS (carcinoma in situ)	2	2.82%
Total	71	100%

Analysis of lipid profiles across benign, premalignant, and malignant gallbladder lesions revealed distinct trends. While mean serum cholesterol was the highest in malignant lesions (210.17 ± 56.98 mg/dL), the difference was not statistically significant (p = 0.3801). Triglyceride levels were significantly elevated in malignant cases (301.75 ± 85.69 mg/dL) compared to premalignant (190.09 ± 90.89 mg/dL) and benign lesions (189.40 ± 128.46 mg/dL; p = 0.0140). HDL levels were the lowest in malignant lesions (37.00 ± 9.05 mg/dL), though not significant (p = 0.3028), with 70% of adenocarcinoma cases exhibiting low HDL (<40 mg/dL). LDL levels showed no significant variation (p = 0.9464). Of the total cohort, three malignant cases had triglyceride values >400 mg/dL and were therefore excluded from LDL correlation analysis due to the limitations of the Friedewald calculation. VLDL was notably higher in malignant lesions (54.82 ± 22.74 mg/dL) compared to benign (34.72 ± 25.35 mg/dL) and premalignant (36.00 ± 19.91 mg/dL) groups, showing a borderline statistical association (p = 0.052) with lesion status (Table 3). These findings suggest an association between malignant transformation and lipid abnormalities, particularly hypertriglyceridemia and elevated VLDL.

**Table 3:** Lipid profile by histopathological nature of lesions, showing the mean ± standard deviation (SD) for key lipid parameters Low-density lipoprotein (LDL) cholesterol was not calculated for three subjects with serum triglyceride levels >400 mg/dL due to the limitation of the Friedewald formula; these cases were excluded from LDL-related analyses. Group comparisons were performed using Welch’s ANOVA due to variance heterogeneity, followed by Games-Howell post-hoc tests where applicable. Sample sizes (n) reflect exclusions as noted above for LDL values. Hypercholesterolemia: S. cholesterol >200 mg/dL; Hypertriglyceridemia: triglyceride >150 mg/dL; High LDL: LDL >100 mg/dL; Low HDL: HDL <40 mg/dL; p ≤ 0.05: statistically significant (ANOVA)

Lesion	Benign (n = 48)	Premalignant (n = 11)	Malignant (n = 12)	ANOVA p-value	F-value
S. Cholesterol (Mean ± SD) (mg/dL)	187.19±51.04	189.91±43.16	210.17±56.98	0.3801	0.981
Triglyceride (Mean ± SD) (mg/dL)	189.40±128.46	190.09±90.89	301.75±85.69	0.014	4.548
HDL (Mean ±SD) (mg/dL)	41.88±9.19	40.55±12.43	37.00±9.05	0.3028	1.216
LDL (Mean ±SD) (mg/dL)	107.79±42.31	112.09±41.92	110.33±41.48	0.9464	0.055

**Figure 2:**
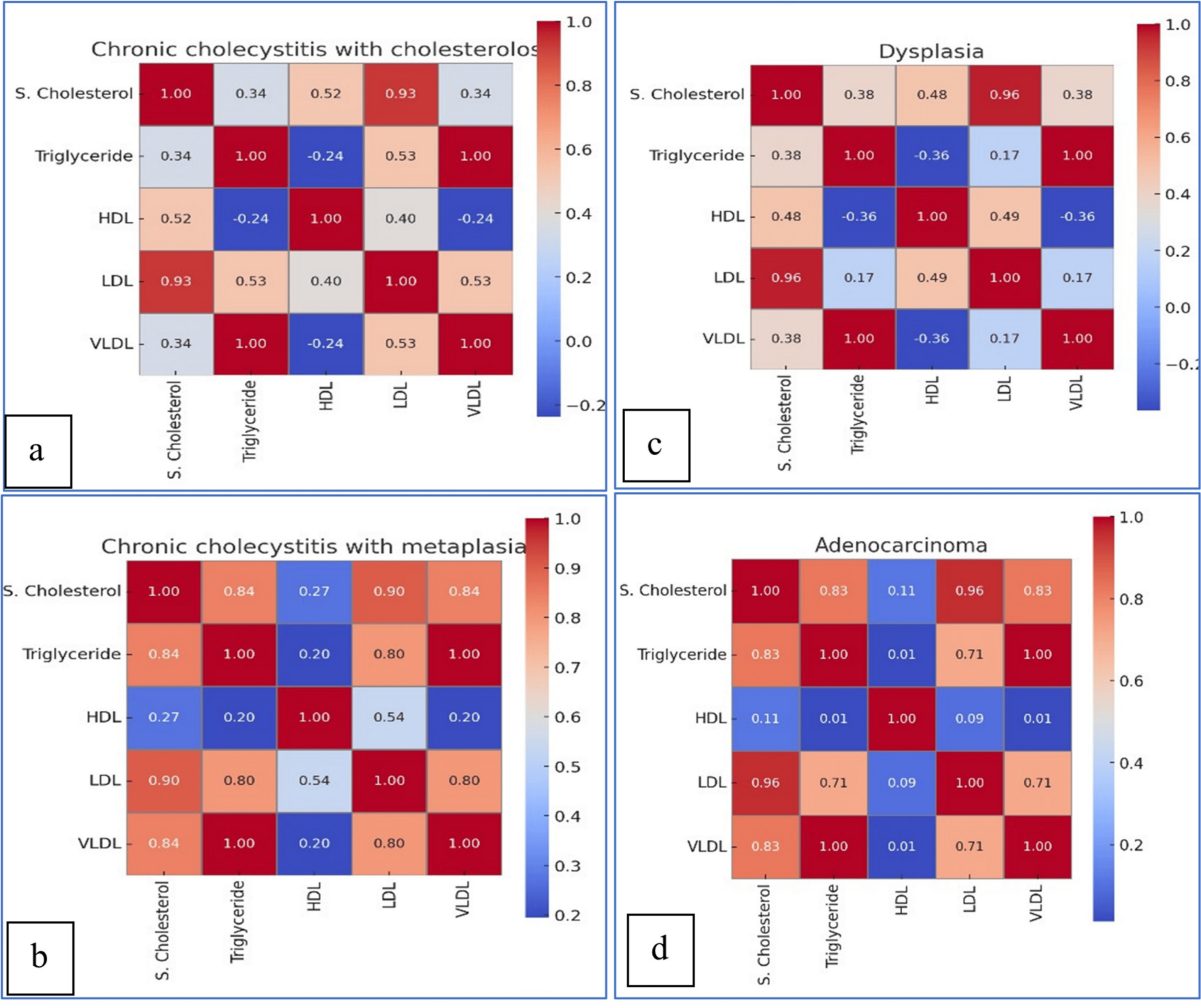
Heatmap visualization showing the correlation between lipid profile parameters (S. cholesterol, triglyceride, HDL, LDL, and VLDL) using Pearson's coefficient (r) for (a) cholecystitis with cholesterolosis, (b) cholecystitis with metaplasia, (c) dysplasia, and (d) adenocarcinoma of the gall bladder Each heatmap illustrates: positive correlations (0 < r ≤ 1) (🔴 red) and negative correlations (-1 ≤ r < 0) (🔵 blue) HDL: high-density lipoprotein; LDL: low-density lipoprotein; VLDL: very-low-density lipoprotein

Correlation analyses revealed striking patterns of lipid dysregulation. The triglyceride-VLDL axis showed perfect correlation across all lesions (r = 1.0). In chronic cholecystitis with cholesterolosis, serum cholesterol showed a very strong correlation with LDL (r = 0.929), while triglycerides were strongly linked to VLDL (r = 1.000) and inversely related to HDL (r = -0.237) (Table 4, Figure 2a). Gallbladder dysplasia exhibited similar trends, with serum cholesterol strongly associated with LDL (r = 0.963) and triglycerides again perfectly correlated with VLDL (r = 1.000), alongside a negative HDL-triglyceride relationship (r = -0.364) (Table 5, Figure 2c). In adenocarcinoma, serum cholesterol was strongly tied to LDL (r = 0.960) and triglycerides/VLDL (r = 0.827), with triglycerides also correlating with LDL (r = 0.714) (Table 6, Figure 2d). Notably, HDL showed minimal associations in malignancies, suggesting metabolic decoupling in advanced disease. These findings highlight lesion-specific lipid interactions, particularly the dominant role of LDL and VLDL in malignant transformation while underscoring the progressive loss of HDL’s protective correlation in cancer.

**Table 4:** Correlation between lipid profile parameters with cholesterolosis using Pearson's coefficient HDL: high-density lipoprotein; LDL: low-density lipoprotein; VLDL: very-low-density lipoprotein

Variables	S. Cholesterol	TG	HDL	LDL	VLDL
S. Cholesterol	1.000	0.342	0.524	0.929	0.342
Triglyceride		1.000	-0.237	0.529	1.000
HDL			1.000	0.399	-0.237
LDL				1.000	0.529
VLDL					1.000

**Table 5:** Correlation between lipid profile parameters with dysplasia using Pearson's coefficient HDL: high-density lipoprotein; LDL: low-density lipoprotein; VLDL: very-low-density lipoprotein

Variables	S. Cholesterol	TG	HDL	LDL	VLDL
S. Cholesterol	1.000	0.383	0.484	0.963	0.383
Triglyceride		1.000	-0.364	0.172	1.000
HDL			1.000	0.490	-0.364
LDL				1.000	0.172
VLDL					1.000

**Table 6:** Correlation between lipid profile parameters with adenocarcinoma using Pearson's coefficient LDL values exclude three samples with triglycerides >400 mg/dL, where the Friedewald calculation was not applicable. Correlation coefficients calculated using Pearson’s method. Significant correlations were discussed in the text. HDL: high-density lipoprotein; LDL: low-density lipoprotein; VLDL: very-low-density lipoprotein

Variables	S. Cholesterol	TG	HDL	LDL	VLDL
S. Cholesterol	1.000	0.827	0.112	0.960	0.827
Triglyceride		1.000	0.012	0.714	1.000
HDL			1.000	0.090	0.012
LDL				1.000	0.714
VLDL					1.000

The analysis of lipid profiles across different histopathological diagnoses revealed notable variations, particularly in triglycerides and VLDL levels. Adenocarcinoma cases showed the highest mean serum cholesterol (219.4 ± 58.08 mg/dL), though differences were not statistically significant (p = 0.2522). Triglycerides varied significantly (p = 0.0079), with the highest levels in adenocarcinoma (302.3 ± 94.67 mg/dL) and CIS (299.0 ± 9.90 mg/dL). HDL levels remained consistent across groups (p = 0.6025), while LDL was the highest in chronic cholecystitis with cholelithiasis (125.1 ± 38.91 mg/dL), though not significant (p = 0.4294). VLDL exhibited significant differences (p = 0.0394), with CIS having the highest levels (79.5 ± 26.16 mg/dL) (Table 7).

**Table 7:** Mean ± standard deviation (SD) of lipid parameters across different histopathological diagnoses of gall bladder lesions LDL cholesterol was not calculated for three subjects with serum triglyceride levels >400 mg/dL due to the limitation of the Friedewald formula; these cases were excluded from LDL-related analyses. Group comparisons were performed using Welch’s ANOVA due to variance heterogeneity, followed by Games-Howell post-hoc tests where applicable. Sample sizes (n) reflect exclusions as noted above for LDL values. Hypercholesterolemia: serum cholesterol >200 mg/dL; Hypertriglyceridemia: triglycerides >150 mg/dL; High LDL: LDL >100 mg/dL; Low HDL: HDL <40 mg/dL; p ≤ 0.05 considered statistically significant CIS: carcinoma in situ; HDL: high-density lipoprotein; LDL: low-density lipoprotein; VLDL: very-low-density lipoprotein

Histopathological Diagnosis	Chronic cholecystitis	Chronic cholecystitis with cholelithiasis	Chronic cholecystitis with cholesterolosis	Xanthogranulomatous cholecystitis	Chronic cholecystitis with metaplasia	Dysplasia	CIS	Adenocarcinoma	ANOVA p-value	F-value
S. Cholesterol (Mean ± SD) (mg/dL)	177.0 ± 57.27	196.0 ± 47.73	208.17 ± 44.34	186.57 ± 40.31	161.22 ± 59.01	189.91 ± 43.16	164.0 ± 15.56	219.4 ± 58.08	0.2522	1.099
Triglyceride (Mean ± SD) (mg/dL)	152.7 ± 86.65	153.8 ± 95.00	222.17 ± 137.65	298.71 ± 169.85	141.0 ± 112.63	190.09 ± 90.89	299.0 ± 9.90	302.3 ± 94.67	0.0079	3.049
HDL (Mean ± SD) (mg/dL)	45.0 ± 8.50	40.6 ± 9.49	43.33 ± 11.37	38.71 ± 9.07	40.33 ± 6.71	40.55 ± 12.43	31.5 ± 0.71	38.1 ± 9.59	0.6025	0.716
LDL (Mean ± SD) (mg/dL)	104.7 ± 48.13	125.1 ± 38.91	119.75 ± 37.32	92.43 ± 35.75	88.0 ± 45.65	112.09 ± 41.92	81.0 ± 4.24	116.2 ± 43.26	0.4294	0.851
VLDL (Mean ± SD) (mg/dL)	29.3 ± 18.07	28.6 ± 20.16	33.9 ± 13.14	55.29 ± 39.76	32.44 ± 30.83	36.0 ± 19.91	79.5 ± 26.16	49.33 ± 19.35	0.0394	1.987

## Discussion

The present study demonstrates a strong correlation linking dysregulated lipid metabolism to the progression of gallbladder lesions from benign to malignant states. Our findings support an association between malignant and premalignant gallbladder lesions and distinct lipid profile abnormalities, particularly marked hypertriglyceridemia and elevated VLDL levels, which correlate with histopathological progression. These observations align with emerging evidence suggesting that metabolic dysregulation may play an important role in gallbladder carcinogenesis, offering new insights into potential diagnostic and therapeutic approaches for this aggressive malignancy. Several studies have reported conflicting associations between serum lipid parameters and cancer risk, with some demonstrating null or even inverse relationships. For example, Ganjali et al. [23] discussed evidence that certain populations with lower LDL cholesterol levels may not have a correspondingly reduced cancer risk. Some studies have even observed inverse trends between LDL and cancer incidence, suggesting that low LDL might sometimes be a marker, rather than a cause, of underlying malignancy or pro-inflammatory states. This highlights the complexity and heterogeneity in the relationship between lipid profiles and cancer risk and underscores the need for further large-scale, prospective investigations to clarify these associations.

It is important to acknowledge that several unmeasured metabolic variables - including BMI, glycemic control (diabetes mellitus), and use of lipid-lowering agents, such as statins - could confound the observed associations between serum lipid profiles and gallbladder lesion progression. Both obesity and diabetes are recognized to alter lipid metabolism and promote systemic inflammation, thereby increasing the risk of various cancers, including biliary tract malignancies. Additionally, statin therapy has been shown in meta-analyses to reduce the risk of biliary tract cancers, likely due to both lipid-lowering and anti-inflammatory effects. Thus, in our study, lack of systematic adjustment for these factors may have introduced confounding, where differences in lipid profiles could in part reflect these underlying metabolic states or medication effects rather than a direct association with neoplastic transformation. Future studies should systematically record and adjust for these variables to clarify the independent role of lipid metabolism in gallbladder cancer risk [24,25].

The demographic patterns observed in our study, with peak incidence in the 51-60-year age group (26.76%, n = 19) and striking female predominance (77.46%, n = 55), corroborate global epidemiological trends reported by Rawla et al. and Mishra et al. [26,27]. The age-dependent progression from benign (mean age: 46.0 years) to premalignant (53.4 years) and malignant lesions (55.1 years) supports the concept of cumulative exposure to metabolic risk factors in gallbladder carcinogenesis. The high prevalence of chronic inflammatory conditions (chronic cholecystitis with cholesterolosis, 16.9%, n = 12; with cholelithiasis, 14.1%, n = 10) preceding dysplastic (15.5%, n = 11) and malignant changes (adenocarcinoma, 14.1%, n = 10: CIS, 2.8%, n = 2) reinforces the inflammation-dysplasia-carcinoma sequence proposed by Seretis et al. [28], particularly in gallstone-associated pathology.

Our most significant finding was the marked elevation of triglyceride levels in malignant lesions (301.75 ± 85.69 mg/dL vs. 189.40 ±128.46 mg/dL in benign, p = 0.014), with 100% of adenocarcinoma cases (n = 10; 95% CI: (69.2%, 100%)) meeting the criteria for hypertriglyceridemia. This strongly supports the hypothesis proposed by Caligiuri et al. that triglyceride-rich lipoproteins promote carcinogenesis through oxidative stress and chronic inflammatory pathways [29]. The parallel elevation of VLDL in malignant cases (54.82 ± 22.74 mg/dL, p = 0.052) and its perfect correlation with triglycerides (r = 1.0) across all lesions suggests VLDL may serve as both a metabolic marker and potential contributor to tumor progression by providing fatty acids for membrane biosynthesis, as suggested by Ganjali et al. [23].

The progressive lipid abnormalities observed - from benign through premalignant to malignant lesions - mirror the histological continuum of gallbladder carcinogenesis. While the total cholesterol and LDL showed non-significant trends, the consistent depression of HDL in malignant lesions (37.00 ± 9.05 mg/dL, p = 0.303) suggests that loss of protective anti-inflammatory effects may facilitate tumor progression, consistent with findings by Patel et al. [30]. Although lower HDL and LDL levels were observed in malignant lesions, these differences did not consistently achieve statistical significance and should be interpreted cautiously. Their potential utility as biomarkers for early detection remains speculative and requires validation in future, larger studies. The particularly strong triglyceride correlation provides mechanistic support for the metabolic theory of carcinogenesis proposed by Riscal et al. [31], suggesting that triglyceride metabolites may directly stimulate epithelial proliferation.

While our findings suggest that serum lipid profiling - particularly triglycerides and VLDL - may be promising for risk stratification, we acknowledge that no definitive diagnostic cut-off or risk algorithm can yet be recommended. Future, larger studies are required to validate these findings and define actionable thresholds. The distinct metabolic signature of malignant lesions (concurrent hypertriglyceridemia, elevated VLDL, and low HDL) may aid in early detection, as emphasized by Sun et al. [10]. The high prevalence of dyslipidemia in premalignant lesions (54.5% hypertriglyceridemia in dysplasia) suggests that metabolic interventions during these stages might potentially alter disease progression, though this requires prospective validation.

This study represents the first Indian cohort analysis encompassing benign, premalignant, and malignant gallbladder lesions with uniform histopathological confirmation and standardized serum lipid assessments. The biological plausibility of our findings, supported by alignment with extant literature, underscores their potential clinical relevance.

Our study has several important limitations that warrant consideration. These results must be interpreted with caution, considering the cross-sectional design, which precludes causal inferences. Although we tested and accounted for statistical assumptions by employing Welch’s ANOVA when variance heterogeneity was detected, the modest sample size - particularly in the malignant (n =12) and carcinoma in situ (n = 2) subgroups - may limit statistical power and precision. While we employed rigorous histopathological classification performed independently by two blinded pathologists with consensus resolution to minimize design misclassification, some potential for diagnostic overlap or subjective interpretation remains inherent in lesion categorization. Additionally, the absence of systematic data collection on metabolic confounders, such as BMI, diabetes status, and pharmacological lipid-lowering interventions, may have influenced serum lipid associations. The lack of statistically robust associations for HDL and LDL, coupled with the exploratory nature of our study design, limits the extent to which these findings can be translated into clinical recommendations for early detection. Our conclusions regarding these lipids should be viewed as preliminary. The modest sample size and single-center nature represent constraints that should motivate further validation in larger, multicenter cohorts. Despite these constraints, we believe that the study provides valuable preliminary evidence linking serum lipid abnormalities with gallbladder lesion progression and highlights the need for prospective, longitudinal studies incorporating confounder adjustment and mechanistic evaluation to substantiate and expand upon our findings. Our findings significantly advance understanding of the metabolic basis of gallbladder carcinogenesis and highlight the potential clinical utility of lipid profiling in managing this aggressive malignancy. Future research should explore molecular mechanisms linking specific lipid metabolites to proliferative signaling pathways and investigate whether triglyceride-lowering interventions can modify cancer risk in high-risk populations.

## Conclusions

This study demonstrates a strong correlation between dysregulated lipid metabolism and the progression of gallbladder lesions from benign to malignant states, with hypertriglyceridemia and elevated VLDL emerging as key metabolic hallmarks of carcinogenesis. While serum lipid profile changes may be associated with the progression of gallbladder lesions, the current cross-sectional evidence is insufficient to establish HDL and LDL as reliable markers for early detection or risk stratification. Prospective studies are necessary to determine their clinical utility. Nevertheless, the progressive lipid derangements across the histopathological spectrum underscore the potential utility of serum lipid profiling - particularly triglycerides and VLDL - as a non-invasive adjunct for early detection and risk stratification in gallbladder cancer and lay the groundwork for future research into metabolic interventions in high-risk populations. While the cross-sectional design precludes causal inferences, the progressive lipid derangements observed across the histological spectrum (benign → premalignant → malignant) underscore the potential of metabolic interventions in gallbladder cancer prevention.

As this was a single-center cohort from northern India, the generalizability of our findings to other populations - especially those with differing dietary habits, genetic backgrounds, or patterns of gallbladder disease - may be limited. Multicenter, multi-ethnic studies are needed to extend the external validity. Future research should explore targeted lipid-modulating strategies and their impact on disease progression, offering new avenues for improving outcomes in this aggressive malignancy with limited therapeutic options.

## References

[1] Sandler RS, Everhart JE, Donowitz M (2002). The burden of selected digestive diseases in the United States. Gastroenterology.

[2] Manu K, Srivastava D, Singh A (2021). Spectrum of histopathological lesions of gallbladder. IJCMR.

[3] Wrenn SM, Callas PW, Abu-Jaish W (2017). Histopathological examination of specimen following cholecystectomy: are we accepting resect and discard?. Surg Endosc.

[4] Siegel RL, Miller KD, Jemal A (2017). Cancer statistics, 2017. CA Cancer J Clin.

[5] Cereda S, Belli C, Reni M (2012). Adjuvant treatment in biliary tract cancer: to treat or not to treat?. World J Gastroenterol.

[6] Lai CH, Lau WY (2008). Gall bladder cancer - a comprehensive review. Surgeon.

[7] de Groen PC, Gores GJ, LaRusso NF, Gunderson LL, Nagorney DM (1999). Biliary tract cancers. N Engl J Med.

[8] Roa I, Araya JC, Villaseca M, De Aretxabala X, Riedemann P, Endoh K, Roa J (1996). Preneoplastic lesions and gallbladder cancer: an estimate of the period required for progression. Gastroenterology.

[9] Rahnemai-Azar AA, Weisbrod A, Dillhoff M, Schmidt C, Pawlik TM (2017). Intrahepatic cholangiocarcinoma: molecular markers for diagnosis and prognosis. Surg Oncol.

[10] Sun L, Ji X, Wang D (2020). Integrated analysis of serum lipid profile for predicting clinical outcomes of patients with malignant biliary tumor. BMC Cancer.

[11] Kiran RP, Pokala N, Dudrick SJ (2007). Incidence pattern and survival for gallbladder cancer over three decades--an analysis of 10301 patients. Ann Surg Oncol.

[12] Sasatomi E, Tokunaga O, Miyazaki K (2000). Precancerous conditions of gallbladder carcinoma: overview of histopathologic characteristics and molecular genetic findings. J Hepatobiliary Pancreat Surg.

[13] Mukhopadhyay S, Landas SK (2005). Putative precursors of gallbladder dysplasia: a review of 400 routinely resected specimens. Arch Pathol Lab Med.

[14] Strom BL, Maislin G, West SL (1990). Serum CEA and CA 19-9: potential future diagnostic or screening tests for gallbladder cancer?. Int J Cancer.

[15] Jayaraman S, Jarnagin WR (2010). Management of gallbladder cancer. Gastroenterol Clin North Am.

[16] Prabhu RS, Hwang J (2017). Adjuvant therapy in biliary tract and gall bladder carcinomas: a review. J Gastrointest Oncol.

[17] Ben-Josef E, Guthrie KA, El-Khoueiry AB (2015). SWOG S0809: a phase II intergroup trial of adjuvant capecitabine and gemcitabine followed by radiotherapy and concurrent capecitabine in extrahepatic cholangiocarcinoma and gallbladder carcinoma. J Clin Oncol.

[18] Hsing AW, Gao YT, Han TQ (2007). Gallstones and the risk of biliary tract cancer: a population-based study in China. Br J Cancer.

[19] Andreotti G, Chen J, Gao YT (2008). Serum lipid levels and the risk of biliary tract cancers and biliary stones: a population-based study in China. Int J Cancer.

[20] Thijs C, Knipschild P, Brombacher P (1990). Serum lipids and gallstones: a case-control study. Gastroenterology.

[21] Xie H, Heier C, Kien B (2020). Adipose triglyceride lipase activity regulates cancer cell proliferation via AMP-kinase and mTOR signaling. Biochim Biophys Acta Mol Cell Biol Lipids.

[22] Gupta A, Gupta S, Rajput D (2021). Expression and clinicopathological correlation of Ki-67 in gallbladder carcinoma. J Carcinog.

[23] Ganjali S, Banach M, Pirro M, Fras Z, Sahebkar A (2021). HDL and cancer - causality still needs to be confirmed? Update 2020. Semin Cancer Biol.

[24] Cheung KS, Yeung YWM, Wong WS, Li B, Seto WK, Leung WK (2023). Statins associate with lower risk of biliary tract cancers: a systematic review and meta‐analysis. Cancer Med.

[25] Pliszka M, Szablewski L (2024). Associations between diabetes mellitus and selected cancers. Int J Mol Sci.

[26] Rawla P, Sunkara T, Thandra KC, Barsouk A (2019). Epidemiology of gallbladder cancer. Clin Exp Hepatol.

[27] Mishra K, Behari A, Shukla P (2021). Risk factors for gallbladder cancer development in northern India: a gallstones-matched, case-control study. Indian J Med Res.

[28] Seretis C, Lagoudianakis E, Gemenetzis G, Seretis F, Pappas A, Gourgiotis S (2014). Metaplastic changes in chronic cholecystitis: implications for early diagnosis and surgical intervention to prevent the gallbladder metaplasia-dysplasia-carcinoma sequence. J Clin Med Res.

[29] Caligiuri A, Becatti M, Porro N (2023). Oxidative stress and redox-dependent pathways in cholangiocarcinoma. Antioxidants (Basel).

[30] Patel KK, Kashfi K (2022). Lipoproteins and cancer: the role of HDL-C, LDL-C, and cholesterol-lowering drugs. Biochem Pharmacol.

[31] Riscal R, Skuli N, Simon MC (2019). Even cancer cells watch their cholesterol!. Mol Cell.

